# Breaking bad news in neurology: assessing training, perceptions, and preparedness among residency programs in Brazil

**DOI:** 10.31744/einstein_journal/2023AO0036

**Published:** 2023-03-24

**Authors:** Thaiza Agostini Córdoba de Lima, Fernando Pereira Bruno, Fernanda Gushken, Luiza Helena Degani-Costa, Natalia Pelizari Novaes

**Affiliations:** 1 Hospital Israelita Albert Einstein São Paulo SP Brazil Hospital Israelita Albert Einstein, São Paulo, SP, Brazil.; 2 Touro College of Osteopathic Medicine Middletown NY USA Touro College of Osteopathic Medicine, Middletown, NY, USA.; 3 Department of Public Health School of Health Sciences and Practice New York Medical College Valhalla NY USA Department of Public Health, School of Health Sciences and Practice, New York Medical College, Valhalla, NY, USA.; 4 Faculdade Israelita de Ciências da Saúde Albert Einstein Hospital Israelita Albert Einstein São Paulo SP Brazil Faculdade Israelita de Ciências da Saúde Albert Einstein, Hospital Israelita Albert Einstein, São Paulo, SP, Brazil.; 5 GHU Paris Psychiatrie et Neurosciences Hôpital Sainte Anne Paris France GHU Paris Psychiatrie et Neurosciences, Hôpital Sainte Anne, Paris, France.

**Keywords:** Communication, Education, Internship and residency, Learning, Mentoring, Neurology, Surveys and questionnaires

## Abstract

**Objective:**

We aimed to evaluate how breaking bad news training was implemented in neurology residency programs in Brazil and to assess the perception and preparedness of trainees and program directors.

**Methods:**

We performed a cross-sectional descriptive study. Neurology trainees and program directors were recruited from the Brazilian Academy of Neurology registry through convenience sampling. Participants answered a survey evaluating the breaking bad news training at their institution and their preparedness and perception towards the topic.

**Results:**

We collected 172 responses from 47 neurology institutions from all five socio-demographic regions of Brazil. More than 77% of trainees were dissatisfied with their breaking bad news training, and around 92% of program directors believed their programs required substantial improvement. Approximately 31% of neurology trainees reported never having a lecture about communicating bad news, 66% reported never having a simulated training, and nearly 61% never received feedback regarding their communication abilities. Moreover, 59% of program directors acknowledged that feedback was not a standard practice and nearly 32% reported the absence of any specific training.

**Conclusion:**

This study suggested that the breaking bad news training in neurology residencies across Brazil is deficient and highlighted challenges to achieve this core competency. Program directors and trainees recognized the importance of the topic, and program directors acknowledged that many factors hinder the ability to implement formal training. Given the relevance of such a skill to patient care, every effort should be made to provide structured training opportunities during residency.

## Highlights

• Neurology trainees and program directors recognized a lack of structured breaking bad news training.

• Program directors reported that many factors hinder the implementation of breaking bad news education.

• Trainees felt capable of breaking bad news, but most did not have lectures, simulations, nor feedback.

• Trainees acknowledged negative feelings when breaking bad news, including sadness and helplessness.

**Figure:**
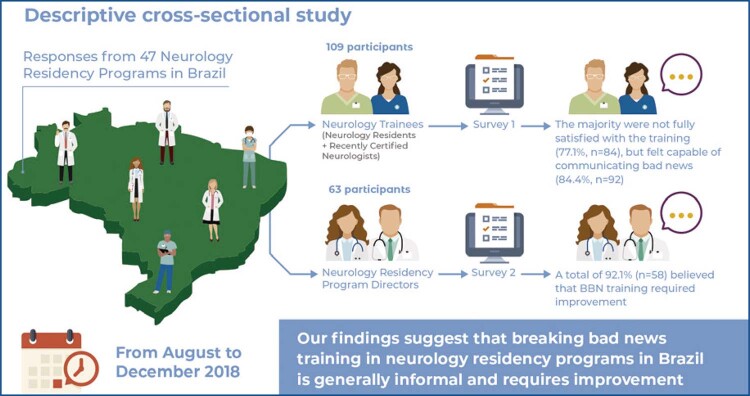


## INTRODUCTION

Defining bad news is complicated, as it is subjective to individual perceptions and beliefs.^( 1 )^ In medicine, bad news refers to something that may negatively alter one’s view of their wellbeing or future.^( 2 )^ Doctors face the challenge of communicating bad news in their daily lives.

Developing communication skills is essential when establishing successful doctor-patient relationships. Adopting effective communication in healthcare has demonstrated an increase in patient satisfaction,^( 3 )^a decrease in malpractice claims,^( 4 )^ and more importantly, a positive impact on patient-centered outcomes, such as therapeutic adherence,^( 5 )^functional status, and mental health.^( 3 )^Therefore, physicians must be trained in communication skills.

Over the past decades, the demand to define a physician’s expected core competencies has increased. Core competencies are a way to encourage programs to work towards a standard set of goals to standardize the major expectations of the medical curricula,^( 6 - 8 )^ even when the educational methodology varies by medical school and residency program. Oral communication is a featured competency, which, combined with social skills and cultural competence, is essential to secure the skillset of breaking bad news (BBN).

Regarding communication skills to deliver bad news as essential in medical training is critical, given that no evidence has suggested that life experiences alone are enough to improve such communication skills.^( 9 )^ In this context, several protocols have been created to help guide physicians through the challenging process of communication skills training. The most widely known system is the SPIKES protocol, a well-established, six-step protocol for conveying bad news.^( 10 )^Considered as one of the gold-standards of BBN, the SPIKES protocol allows physicians to accomplish the four most relevant objectives when delivering bad news: i) gather information from the patient, ii) transmit the medical information, iii) provide support to the patient, and iv) elicit the patient’s collaboration in developing a strategy or treatment plan for the future.^( 10 )^ This protocol has since been adapted to other areas of medicine, including obstetrics and gynecology, radiology, emergency medicine, and critical care. However, no specific protocol exists for neurology.

In neurology, BBN is a frequent occurrence. Neurologists deal with many diseases with a poor prognosis, frequently with an incurable and progressive nature that can result in chronic disability or death.^( 11 )^ Despite the frequency and complexity of difficult conversations in neurology, the training and evaluation of communication skills are primarily informal.^( 12 )^Gaps in current training and opportunities for growth for neurology trainees regarding their understanding of communication and palliative care principles have recently been identified.^( 13 )^Therefore, guaranteeing that neurologists feel comfortable and proficient in BBN should be an important milestone of their training.

Training strategies should be primarily based on lectures, practice in simulated or real-life settings, and serial structured feedback.^( 14 )^The effectiveness of structured training has been demonstrated in several studies. For example, Liénard et al. demonstrated how structured training improved patient-centered communication skills.^( 15 )^ Meanwhile, Dosanjh et al.^( 16 )^ demonstrated that the lack of emotional support for residents is an obstacle when communicating bad news.

In Brazil, the Ministry of Education serves as the centralized governance that proposes national curriculum guidelines for medical schools and residency programs. In 2014, the Ministry of Education published updates to the National Curricular Guidelines, providing the first mention of communication as a core competency for medical students, highlighting “empathy, sensitivity and interest, preserving the confidentiality, understanding, autonomy, and security of the person under care.”^( 17 )^ Since the 1984 edition, the Brazilian Code of Medical Ethics, established by the Federal Council of Medicine (CFM), declares that physicians are “forbidden from not telling the truth to patients about their diagnosis, prognosis, treatment risks, and treatment goals.”^( 18 , 19 )^ Consequently, although the Ministry of Education directives did not directly address BBN, the CFM does determine that physicians have an ethical and legal duty to break bad news to their patients and their families.

## OBJECTIVE

Given the relevance of the core competency in communication and heterogeneity on how breaking bad news might be offered in different residency programs, we aimed to evaluate how breaking bad news training was implemented in neurology residency programs in Brazil. Additionally, we further assessed the perceptions and preparedness of trainees and program directors on delivering bad news.

## METHODS

We performed a descriptive cross-sectional study using data collected from August to December of 2018. The population of interest was classified into two different groups: PDs and neurology trainees. Program Directors were defined as faculty with curricular oversight in the accredited neurology programs of Brazil. The neurology trainees included neurology residents and recently certified neurologists (RCNs), defined as first-time board-certified neurologists between 2015 and 2017. In Brazil, neurology residency consists of three years of training: the first year in internal medicine (post-graduate year 1, PGY-1) and two additional years in neurology (PGY-2 and PGY-3). As such, only PGY-2 and PGY-3 residents were recruited for the study, as they were in the neurology-specific stage of their training.

The research instrument consisted of two sets of electronic questionnaires built on a Research Electronic Data Capture (REDCap) survey platform.^( 20 , 21 )^ These questionnaires were composed of multiple-choice and open-ended questions meant to evaluate participants’ perceptions and competence on BBN, including areas for improvement in their training. The questionnaire was tailored to the aims of our study and not adapted from previous questionnaires. A formal statistical analysis is not presented herein, as we performed qualitative descriptive research. All answers were anonymous, and formal consent was obtained electronically. Finally, the data were gathered after the project was approved by the *Hospital Israelita Albert Einstein* ethics committee and registered under the code CAAE: 93810218.8.0000.0071; # 3.047.929.

When invited to answer the survey, respondents that self-identified as part of the neurology trainee group (PGY-2 and PGY-3 neurology residents, or RCNs) were prompted to answer the questionnaire in Appendix 1, while those that self-identified as part of the PD group were prompted to answer the questionnaire in Appendix 2.

Appendix 1, for neurology trainees, consisted of 21 multiple-choice questions, which assessed BBN training types during residency, satisfaction with training, the importance of the topic, emotions triggered when delivering bad news to patients and family members, communication techniques, and knowledge about the SPIKES protocol.

Appendix 2, for neurology residency PDs, consisted of five multiple-choice questions and one open-ended question, which assessed types of BBN training in the residency program, the perceived need for improvements, importance of the topic, and potential barriers to improving BBN training in their program.

The national recruitment process consisted of convenience sampling of all eligible physicians, trainees, and PDs registered under the e-mail listing service of the Brazilian Academy of Neurology (ABN *- Academia Brasileira de Neurologia* ). In collaboration with the ABN, an e-mail containing a description of our study and a link to the consent form and REDCap questionnaires was sent to all eligible physicians.

According to a medical demographic study published by the *Universidade de São Paulo* (USP) and the *Conselho Regional de Medicina do Estado de São Paulo* (CREMESP)^( 22 )^ in 2018, Brazil had 97 neurology residency programs and 749 neurology residents, 443 of whom were PGY-2 and PGY-3 residents. Between 2015 and 2017, an average of 185 graduating neurologists were trained each year, totaling an estimated 555 possible RCNs in the study period and a possible total of 998 individuals who could constitute the trainee group. Through the ABN e-mailing list, we attempted to recruit all eligible physicians at all 27 of Brazil’s federative units (26 States and 1 Federal District) and all 97 registered neurology programs.

The Instituto Brasileiro de Geografia e Estatística divides the country into five regions (North, Northeast, Central-West, Southeast, and South), based on natural, social, cultural, and economic similarities.^( 23 )^Questionnaires were sent to all regions, and results were organized based on these five regions. The dataset used in this study can be found in the Harvard Dataverse repository.^( 24 )^

### Availability of data and materials

All data has been made available as addendum documents, and the original data is available on a cloud server.^( 25 )^

## RESULTS

### Outcome

The recruitment yielded 172 participants from 47 different institutions in 17 of the 27 federative units, representing all five of the regions of Brazil. A total of 109 participants from 47 institutions self-identified as neurology residents or RCNs and were included as neurology trainees in Group 1 and answered Questionnaire 1. Additionally, 63 participants from 38 institutions self-identified as PDs and were included in Group 2 and answered Questionnaire 2. Regarding sex, 58% of participants were female and 42% were male.

The obtained sample of 109 trainees corresponds to approximately 11% of the estimated 998 available individuals in the trainee group (PGY-2, PGY-3 and RCNs). Responses from trainees were obtained from 47 institutions, and given the 97 neurology residency programs in neurology, that corresponds to 49% of the country’s residency programs. Similarly, responses from PDs were obtained from 38 different institutions, which corresponds to 39% of the country’s residency programs.

**Figure 1:**
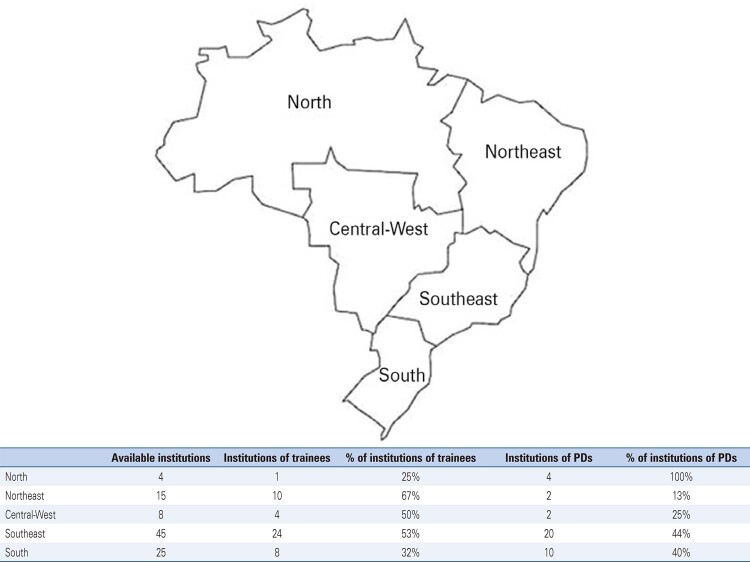
National distribution of Brazilian neurology residency programs grouped by the five socio-demographic regions of Brazil The figure shows the number of programs per region and the number of programs represented by neurology trainees and PDs. (Trainees: PGY-2 and PGY-3 neurology residents and RCNs. PDs: faculty involved with the curricular determinations of that particular neurology program).

The state of São Paulo represented the largest number of participants, with 54.4% (n=55) of neurology trainees and 36.5% (n=23) of PDs, followed by the state of Minas Gerais, with 10% (n=11) of neurology trainees and 17.4% (n=11) of PDs. The other participating states included Rio Grande do Sul, Bahia, Ceará, Distrito Federal, Paraná, Rio de Janeiro, Goiás, Santa Catarina, Amazonas, Mato Grosso do Sul, Pernambuco, Rio Grande do Norte, Sergipe, Alagoas, and Pará. We compiled the institutional results by region, demonstrating the distribution and representation of our data for each group ( Figure 1 ).

PDs: program directors.

#### Group 1: Neurology trainees

#### Methods and perceptions

Within Group 1, all respondents considered BBN training important (100%, n=109), and the majority felt capable of communicating bad news (84.4%, n=92). However, 77.1% (n=84) of participants were not fully satisfied with the BBN training provided during residency ( Table 1 ).

**Table 1:** Summary results from Group 1 - Neurology Trainees

Questionnaire 1 - Residents and Recently Certified Neurologists (part 1 of 2)	n (%)
Sex	
Male	46 (42.20)
Female	63 (57.80)
Age, average	30.4
Do you feel capable of communicating bad news?	
Yes	92 (84.40)
No	17 (15.60)
Are you satisfied with the training on the communication of bad news in your residency program?	
Completely satisfied	25 (22.94)
Partially satisfied	61 (55.96)
Not satisfied	23 (21.10)
Do you consider the training on the communication of bad news to be important?	
Yes	109 (100.00)
No	0 (0.00)
How many times have you had a lecture on the communication of bad news?	
Never	34 (31.19)
Once	40 (36.70)
At least twice	35 (32.11)
How many times have you participated in simulations (role-playing with actors or residents)?	
Never	72 (66.06)
Once	22 (20.18)
At least twice	15 (13.76)
During residency, how many times did you experience real situations involving the communication of bad news?	
Never	2 (1.83)
Once	0 (0.00)
At least twice	107 (98.17)
During residency, how many times did you receive feedback on your performance of bad news communication?	
Never	66 (60.55)
Once	18 (16.51)
At least twice	25 (22.94)
Do you know the SPIKES protocol?	
Yes	64 (58.72)
No	45 (41.28)
**Questionnaire 1 - Residents and Recently Certified Neurologists (part 2 of 2)**	**n (%)**
Think of the last instance of communication of bad news that you participated in: Was a particular technique used when delivering bad news?	
Yes	50 (45.87)
No	59 (54.13)
Where in the hospital was the news given?	
Bedside	9 (8.26)
Hall	29 (26.61)
Reserved room	71 (65.14)
What are your feelings when delivering bad news?	
No bad feelings, I am already used to this situation	19 (17.43)
Insecurity	23 (21.10)
Fear	10 (9.17)
Anguish	28 (25.69)
Helplessness	36 (33.03)
Sadness	53 (48.62)
Pity	22 (20.18)
Anger/Rage	1 (0.92)
Frustration	21 (19.27)
Choose the factors that hinder your ability to communicate bad news:	n=106
It is hard to find the appropriate words	36 (33.96)
The conversations are emotionally charged in a way that is hard for me	20 (18.87)
These are conversations one cannot prepare for	24 (22.64)
It is hard to balance honesty with the family while trying to salvage their hopes	60 (56.60)
The education level of the family	36 (33.96)
The religious beliefs of the family	19 (17.92)
How do you usually prepare before communicating bad news?	n=108
I mentalize a script to express the ideas	77 (71.30)
I write a list of primary points that need to be communicated	4 (3.70)
I discuss with the faculty	15 (13.89)
I discuss with another resident	7 (6.48)
I revise protocols proposed by the literature	4 (3.70)
I do not prepare; my activities in the residency are so demanding that I do not have time for any type of specific preparation	25 (23.15)
Do you ask about the patient’s perspectives or points of view?	
Yes	91 (83.49)
No	18 (16.51)
Do you attempt to use plain language, allowing for clear and objective explanations?	
Yes	108 (99.08)
No	1 (0.92)
Do you offer the information gradually, allowing it to be retained (approach possible diagnosis, therapeutic plan, and prognosis separately)?	
Yes	98 (89.91)
No	11 (10.09)
Do you ask if the patient has any questions after your explanation?	
Yes	109 (100.00)
No	0 (0.00)
Do you repeat and summarize the primary points that were explained?	
Yes	79 (72.48)
No	30 (27.52)

Concerning the BBN training method in their residency program, 31.2% (n=34) of participants reported never having a specific lecture on the topic, and 66.1% (n=72) answered that they never participated in simulations for BBN. Finally, 60.6% (n=66) of residents reported that they never received feedback on their performance when communicating bad news ( Table 1 ).

Although most participants reported that they had given bad news at least twice during residency (98.2%, n=107), only 45.9% (n=50) said that they had used some sort of communication technique in their last experience. Moreover, 41.3% (n=45) of participants were not familiar with the SPIKES protocol.

## Knowledge and use of BBN techniques among trainees

Regarding self-reported emotions triggered when delivering bad news, most participants (82.6%, n=90) reported some sort of negative feeling ( Table 1 ), including sadness (48.6%, n=53), helplessness (33%, n=36), anguish (25.7%, n=28), insecurity (21.1%, n=23), pity (20.2%, n=22), frustration (19.3%, n=21), fear (9.2%, n=10), and anger/rage (0.9%, n=1).

Upon assessing the perceived challenges in delivering bad news, the results mainly encompassed emotional difficulties or lack of technique. Within the emotional challenges, 56.6% (n=60) of trainees expressed hardships in balancing honesty with the families while trying to salvage their hopes, and 18.9% of trainees expressed that these emotionally charged conversations were hard on them (n=20). Regarding a lack of technique, 34% of trainees (n=36) expressed not finding the right words, and 18.9% of participants (n=20) expressed that they could never be prepared for such conversations. External problems were also selected, including challenges when dealing with the families’ educational level (34%, n=36) or religious beliefs (17.9%, n=19).

### Group 2: PDs

### Methods and perceptions

Within Group 2, 96.8% of the respondents (n=61) believed BBN training was important, and 92.1% (n=58) thought that their programs’ approach to BBN required improvement ( Table 2 ).

Regarding how BBN training was offered in their programs ( Table 2 ), 31.7% of PDs (n=20) reported a lack of specific training. Among the remaining 68.3% of PDs who reported some sort of training, most (57.1%; n=36) stated that BBN training was based on participation in supervised real-life situations, 15.9% (n=10) reported participation in unsupervised real-life situations, and 38.1% (n=24) reported observation of real-life situations. A small proportion of PDs reported providing simulation-based training (6.3%; n=4) or formal classes (9.5%; n=6). Concerning feedback, 58.7% (n =37) admitted this was not standard practice in their programs.

## Challenges

Among the challenges in improving BBN training, the following were listed: the time availability of PDs (38.1%, n=24), time availability of residents (33.3%, n=21), lack of interest of preceptors (28.6%, n=18), and lack of interest of residents (23.8%, n=15). The other challenges listed by PDs (9.5%, n=6) included lack of qualified personnel to perform the training and lack of institutional support ( Table 2 ).

## DISCUSSION

To our knowledge, this was the first study to address BBN training in neurology residency programs in Brazil. In November 2019, in partnership with the National Commission of Resident Physicians and the ABN, the Ministry of Education published a set of core competencies for neurologists, which suggested that doctor-patient communication, including bad news communication techniques, should be an essential requirement in residency training.^( 25 )^ Therefore, our data from 2018 serves as an indispensable baseline that should guide future studies on the topic of BBN training.

In this nationwide survey with residents, RCNs, and PDs, most participants agreed on the importance of BBN training and acknowledged their dissatisfaction with the existing training offered in their program. Unlike international recommendations,^( 7 , 10 )^training in these programs was predominantly informal and primarily based on care delivery experience, without structured feedback. Although residents and RCNs generally felt able to communicate bad news appropriately, the majority denied using any existing technique and reported an emotional toll when delivering bad news. These findings suggest that despite being able to execute the task itself, they could have benefited from formal training on BBN, appropriate feedback, and emotional support after BBN.

Communication skills are not innate, but rather acquired skills that require practice and refinement.^( 9 )^ While complete training must include lectures, practice in simulated or real-life settings, and serial structured feedback,^( 14 )^ our study shows that the neurology residencies of Brazil have not been routinely implementing BBN training. Likewise, more than 60% of RCNs and residents did not recall participating in simulations or receiving feedback during their training.

A notable discrepancy was observed between the responses of trainees and PDs regarding BBN lectures. Nearly 60% of neurology trainees reported having at least one class on the topic, while only 9.5% of PDs reported this as standard practice in their program. Nevertheless, most trainees and PDs reported no simulation-based training during residency. Moreover, many participants considered real-life practice with or without supervision equivalent to training. This perception highlights a frequent misconception that mere observation or full autonomy would be enough to shape behavior. However, observation of experienced professionals is not effective if the environment is not structured and feedback is not provided.^( 26 )^Finally, despite its increased adherence, Objective Structured Clinical Examination (OSCE) is not broadly included in Brazilian programs,^( 27 )^ often due to funding limitations. In countries where OSCE is a national stipulation, the cost of such programs ( *e.g.* , hiring costs of actors serving as standardized patients, technologies to record the student-standardized patient encounter) is usually integrated into the dedicated medical school budget. However, this reality may not be true for many countries, including Brazil. In these instances, programs may consider creative, non-costly alternatives to improve their ability to implement OSCE programs ( *e.g.* : faculty or students who serve as practice volunteers, real-life patient volunteers). As a result, trainees may at times be deprived of a safe practice environment for hands-on training, where an error could be welcomed as a learning opportunity.

Notwithstanding the deficiencies of the curricula of participating programs and the overall dissatisfaction with BBN training at the time of the study, most trainees generally felt able to communicate bad news appropriately. However, because we did not utilize examination tools to evaluate the trainees’ competency in delivering bad news, we could not conclude whether their perception reflected reality. However, a trial with Belgian residents demonstrated that a 40-hour course on BBN and stress management significantly improved patient-centered communication skills compared to no structured training.^( 23 )^ In the study by Liénard et al., trained residents used more open questions, spent more time assessing the simulated patient’s understanding and emotional status, and significantly decreased the amount of technical information provided, favoring plain language.^( 15 )^ Therefore, while the neurology trainees in our study felt capable of appropriately communicating bad news, they could likely improve their communication skills with structured training. This view is further corroborated by the fact that more than half of them did not use any specific technique to deliver bad news.

Among the barriers to implementing structured BBN training, PDs reported limited time and absence of trained professionals that could assist in a more organized approach. Nevertheless, delivering bad news is a core competency to be learned and valued as much as any technical aspect of clinical procedures. Therefore, the transition to an effective training model could begin by training PDs to enable them to impart structured knowledge to residents.

The lack of interest from residents was another barrier to training mentioned by the PDs. This perception, however, conflicts with our results, because all participants from the trainee group considered BBN training important, according to the questionnaire. While we acknowledge a possible selection bias in that someone interested in this topic may be more likely to answer the survey, the possibility that residents’ apparent lack of interest could originate from the absence of training cannot be ignored. Indeed, a Canadian study revealed that a lack of emotional support was an obstacle to communicating bad news, and residents expressed the need to discuss their emotions and performance with preceptors.^( 16 )^ Accordingly, in our study, most trainees reported negative feelings when delivering bad news, revealing what can be interpreted as an unmet need of our neurologists-in-training.

Similarly, lack of time during residency was a problem acknowledged by 23.2% of trainees and 33.3% of PDs. Residency training represents formative years in a young physician’s life, both professionally and emotionally, as they learn to cope with the demanding practical aspects of this career. These findings suggest that programs should reassess the steps they are taking to secure the wellbeing of their trainees, especially when these results are considered in parallel with our findings on the emotional toll taken when delivering bad news.

Nevertheless, our study had some limitations. First, recall bias may have resulted from the self-reporting nature of the study and the absence of a formal curricular assessment of the institutions. For example, some respondents may not have remembered attending classes or receiving feedback. However, the fact that residency PDs overwhelmingly acknowledged the absence of formal BBN training confirms the relevance of the problem. Second, communication techniques may have been taught in medical school rather than during residency. Third, although almost 50% of neurology PDs participated in this study, only 10% of residents and RCNs answered the questionnaire, which may have introduced a level of selection bias, limiting the generalizability of our findings. Lastly, our data were collected in late 2018, before the Brazilian Ministry of Education published BBN as part of its recommended set of core competencies for neurology residents in 2019. However, during the study period, the Ministry of Education had published guidelines for communication, and the CFM had recommendations for BBN. Nevertheless, the trainees and PDs acknowledged issues with meeting these goals, and participants recognized their ability to perform the communication task but stated that expert support in the form of structured training and feedback would have been appreciated. These findings suggest a gap between the guidelines proposed by central governance and the programs’ ability to meet these goals.

Despite its limitations, this study highlighted the possible gaps, barriers, and opportunities in training in neurology residencies, providing data for comparison in future studies. These findings have the potential to motivate neurology residency PDs to organize structured training focused on developing communication skills. Furthermore, this study suggests that where investments in training may still be limited, other non-costly strategies could be developed and implemented, including the use of volunteers.

## CONCLUSION

Our findings suggest that breaking bad news training in neurology residency programs in Brazil is generally informal and requires improvement. Our data should serve as a baseline for future studies. With it, programs can evaluate how to troubleshoot the recognized challenges outlined in this manuscript, and future studies can reassess the programs and compare how possible changes implemented overtime could have impacted the neurology programs. Above all, this study aims to increase awareness among the academic community regarding such deficiencies in neurology residency programs and identify barriers to implementing structured BBN training to design more appropriate programs.

**Table 2:** Summary of responses from Group 2 - Program Directors

Questionnaire 2 - Program Directors	n (%)
1. Institution	
2. What type of training is used for the communication of bad news in your residency program?	
Formal lectures	6 (9.52)
Simulation of real scenarios	4 (6.35)
Observation of real situations	24 (38.10)
Active participation in real situations, with supervision	36 (57.14)
Active participation in real situations, without supervision	10 (15.87)
There is no specific training	20 (31.75)
3. Do the residents in your program usually receive feedback on their ability to communicate bad news?	
Yes	26 (41.27)
No	37 (58.73)
4. Would you like to provide more activities in your program to improve your residents’ training on bad news communication?	
No, because they already have the adequate training	2 (3.17)
No, because I do not consider it a relevant topic	1 (1.59)
No, because I first want to promote changes in other areas of the residency that I find more important	2 (3.17)
Yes, I find the topic important, and I think it requires improvement	58 (92.06)
5. Which factors hinder the implementation of more activities with the objective of training in the communication of bad news?	
Time availability of the residents	21 (33.33)
Time availability of the professors	24 (38.10)
The interest of the residents	15 (23.81)
The interest of the faculty	18 (28.57)
There is no factor	15 (23.81)
Other	6 (9.52)
5a. What other factors hinder the implementation of more activities for training in the communication of bad news?	
“Lack of interest of our institution to promote discussion on this topic”	
“Lack of specific training”	
“Lack of institutional interest and trained personnel for offering the training”	
“Lack of training of the faculty on this subject”	
“There is a need for theoretical fundaments, with trained personnel to teach it”	
“Lack of real cases to practice communication of bad news”	
6. Do you consider the training on the communication of bad news to be important?	
Yes	61 (96.83)
No	2 (3.17)

Results are presented in the supplemental database.

## Appendix 1: Questionnaire answered by neurology trainees



Click here for additional data file.
