# Supplementary material for: Breaking bad news in neurology: assessing training, perceptions, and preparedness among residency programs in Brazil
Source: Einstein (Sao Paulo). 2023 Mar 24;21:eAO0036. doi: 10.31744/einstein_journal/2023AO0036 (PMC10118364; doi:10.31744/einstein_journal/2023AO0036)
Supplement: Supplementary file 1 [file 2317-6385-eins-21-eAO0036-suppl01.pdf]

Appendix 1: Questionnaire answered by neurology trainees

1. Sex
2. Age
3. Institution

Answer the following based on your experience during your residency:

4. Do you feel capable of communicating bad news?
( ) Yes ( ) No
5. Are you satisfied with the training on the communication of bad news offered in your residency program?
( ) Completely satisfied ( ) Partially satisfied ( ) Not satisfied
6. Do you consider the training on the communication of bad news to be important?
( ) Yes ( ) No
7. How many times have you had a lecture on the communication of bad news?
( ) Never ( ) Once ( ) At least twice
8. How many times have you participated in simulations (role-playing with actors or residents)?
( ) Never ( ) Once ( ) At least twice
9. During residency, how many times did you experience real situations involving the communication of bad news?
( ) Never ( ) Once ( ) At least twice
10. During residency, how many times did you receive feedback on your performance of bad news communication?
( ) Never ( ) Once ( ) At least twice
11. Do you know the SPIKES protocol?
( ) Yes ( ) No

Think about the last instance of breaking bad news you participated in:

12. Was a particular technique used when delivering bad news?
( ) Yes, which? _____ ( ) No
13. Where in the hospital was the news given?
( ) Bedside ( ) Hall ( ) Private room
14. What are your feelings when delivering bad news?
( ) No bad feelings, I'm used to this situation.
( ) Insecurity
( ) Fear
( ) Anguish
( ) Helplessness
( ) Sadness
( ) Pity
( ) Anger or rage
( ) Frustration
( ) Others ________
15. Choose the factors that hinder your ability to communicate bad news:
( ) It is hard to find appropriate words
( ) The conversations are emotionally charged in a way that is hard for me
( ) These are conversations one cannot prepare for
( ) It is hard to balance honesty with the family while trying to salvage their hopes
( ) The educational level of the family
( ) The religious beliefs of the family
( ) Other: __________
16. How do you usually prepare for communicating bad news?
( ) I mentalize a script to express the ideas
( ) I write a list of primary points that need to be communicated
( ) I discuss it with the faculty
( ) I discuss it with another resident
( ) I review protocols proposed by the literature
( ) I do not prepare; my activities in the residency are so demanding that I do not have time for any type of specific preparation
( ) Other:___________
17. Based on self-assessment, do you usually perform the following?
Question the patient's perspectives and views:
( ) Yes ( ) No
18. Attempt to use plain language, allowing for clear and objective explanations:
( ) Yes ( ) No
19. Offer the information gradually, allowing it to be retained (approach possible diagnosis, therapeutic plan, and prognosis separately):
( ) Yes ( ) No
20. Ask if the patient has any questions after your explanation:
( ) Yes ( ) No
21. Repeat and summarize the primary points that were explained:
( ) Yes ( ) No

Appendix 2: Questionnaire answered by neurology residency program directors

1. Institution: ___________
2. What type of training is used for the communication of bad news in your residency program?
   ( ) Formal lectures
   ( ) Simulation of real situations
   ( ) Observation of real situations
   ( ) Active participation in real situations, with supervision
   ( ) Active participation in real situations, without supervision
   ( ) There is no specific training
   ( ) Others: ______________________
3. Do the residents in your program usually receive feedback on their ability to communicate bad news?
   ( ) Yes ( ) No
4. Would you like your program to provide more activities to improve your residents' training on bad news communication?
   ( ) No, because I think they already have adequate training
   ( ) No, because I do not consider it a relevant topic
   ( ) No, because I first want to promote changes in other areas of the residency that I find more important
   ( ) Yes, I find the topic important, and I think it requires improvement
5. Which factors hinder the implementation of more activities with the objective of training on the communication of bad news?
   ( ) Time availability of the residents
   ( ) Time availability of the professors
   ( ) The interest of the residents
   ( ) The interest of the faculty
   ( ) There is no factor
   ( ) Other: ______
6. Do you consider training on the communication of bad news to be important?
   ( ) Yes
   ( ) No
